# Addressing food insecurity in rural primary care: a mixed-methods evaluation of barriers and facilitators

**DOI:** 10.1186/s12875-024-02409-1

**Published:** 2024-05-11

**Authors:** Kayla E. Jordanova, Arvind Suresh, Chelsey R. Canavan, Tiffany D’cruze, Alka Dev, Maureen Boardman, Meaghan A. Kennedy

**Affiliations:** 1 Department of Family and Preventive Medicine, University of Utah, 375 Chipeta Way, Suite A, Salt Lake City, UT 84108 USA; 2 Department of Medicine, University of California San Francisco, 505 Parnassus Avenue, M1480, San Francisco, CA 94143 USA; 3 Population Health Department, Dartmouth Hitchcock Medical Center, 1 Medical Center Drive, Lebanon, NH 03766 USA; 4 The Dartmouth Institute for Health Policy and Clinical Practice, Dartmouth College, 1 Medical Center Dr, Lebanon, NH 03756 USA; 5 Geisel School of Medicine at Dartmouth, 1 Rope Ferry Road, Hanover, NH 03755 USA; 6 Department of Community and Family Medicine, Geisel School of Medicine at Dartmouth, 1 Medical Center Drive, Lebanon, NH 03756 USA

**Keywords:** Food insecurity, Primary care, Rural health, Social determinants of health

## Abstract

**Background:**

Food insecurity (FI) is associated with negative health outcomes and increased healthcare utilization. Rural populations face increased rates of FI and encounter additional barriers to achieving food security. We sought to identify barriers and facilitators to screening and interventions for FI in rural primary care practices.

**Methods:**

We conducted a mixed-methods study using surveys and semi-structured interviews of providers and staff members from rural primary care practices in northern New England. Survey data were analyzed descriptively, and thematic analysis was used to identify salient interview themes.

**Results:**

Participants from 24 rural practices completed the survey, and 13 subsequently completed an interview. Most survey respondents (54%) reported their practices systematically screen for FI and 71% reported food needs were “very important” for their patients and communities. Time and resource constraints were the most frequently cited barriers to screening for and addressing FI in practices based on survey results. Interview themes were categorized by screening and intervention procedures, community factors, patient factors, external factors, practice factors, process and implementation factors, and impact of FI screening and interventions. Time and resource constraints were a major theme in interviews, and factors attributed to rural practice settings included geographically large service areas, stigma from loss of privacy in small communities, and availability of food resources through farming.

**Conclusions:**

Rural primary care practices placed a high value on addressing food needs but faced a variety of barriers to implementing and sustaining FI screening and interventions. Strategies that utilize practice strengths and address time and resource constraints, stigma, and large service areas could promote the adoption of novel interventions to address FI.

**Supplementary Information:**

The online version contains supplementary material available at 10.1186/s12875-024-02409-1.

## Background

Food security is defined as “access by all people at all times to enough food for an active, healthy life” [1, 2]. In 2022, 12.8% of U.S. households were food insecure, according to a report from the U.S. Department of Agriculture [1]. Effective screening and interventions can mitigate the negative health outcomes and higher healthcare utilization and costs associated with food insecurity (FI) [3, 4]. Health care organizations can identify and address FI among the patients they serve. However, implementation remains low; a 2019 study evaluating screening for social risk factors found that only 29.6% of physician practices and 39.8% of hospitals screen for FI [5]. Understanding and addressing barriers to FI screening and interventions in healthcare settings is critical to increasing food security among patients.

Primary care settings are well-suited for implementing FI screening and interventions given the longitudinal relationships they have with patients. Several primary care professional societies, including the American Academies of Pediatrics and Family Medicine and the American College of Physicians, recommend integrating FI screening and resource referrals into routine health care [6–8]. Primary care patients agree that FI should be addressed in health care settings, with 84% reporting that FI screening is beneficial [9]. However, several barriers limit screening in primary care, including limited financial resources, time, incentives, reimbursement, and knowledge about FI; complexities regarding referral to services and limited resources to address food insecurity; and concerns about privacy and stigma [10–12]. A survey of pediatricians found that although 80% reported willingness to screen for FI, only 15% implemented screening; the greatest concern was lack of knowledge of how to handle positive screens for FI [13]. Studies have also identified facilitators to FI screening and interventions in primary care settings, including trust between staff and patients, availability of multiple screening modalities (e.g., self-administered on paper or staff-administered), and assistance navigating community resources [10].

Rural practices face additional challenges in addressing food insecurity. Rural households have higher rates of FI than the national average, with 14.7% experiencing FI in 2022 [1]. Yet, physicians are less likely to report screening for FI in rural areas than in urban areas [14]. Rural communities are also challenged by geographical inequality in access to healthy food, as many rural communities have few or no grocery stores, [15] leading to increased transportation costs, higher food prices, and reliance on convenience stores with lower availability and quality of healthful foods [16, 17]. Examples of strategies that rural communities use to improve food access include state funding to support food retailers, [18] farmers markets with vendors that accept Supplemental Nutrition Assistance Program (SNAP), [19] community supported agriculture programs, [20] and food pantries, especially those that offer mobile programs [21].

Rural primary practices also face unique care delivery challenges due to workforce shortages, resulting in larger patient panel sizes [22]. Providers also have a broader scope of responsibilities due to fewer specialist and ancillary care services in the community and serve a larger geographic area with disparities in the availability of community-based resources [22, 23]. Rural health care settings have developed creative solutions to address these challenges, including connecting patients to community-supported agriculture [24], growing food on-site or providing vouchers for local food stores [25], and distributing fresh food onsite or via delivery services [26]. However, little is published about barriers and facilitators to FI interventions. While our team identified lack of transportation and food distribution services as rural barriers to FI screening and interventions in our previous qualitative work with prenatal practices, [27] studies in rural primary care settings are limited. Due to the higher rates of FI in rural households and the unique challenges of rural primary care practices, we sought to identify barriers and facilitators to addressing FI in rural primary care practices in northern New England, USA.

## Methods

We conducted a mixed-methods study consisting of a structured survey and semi-structured interviews to evaluate barriers and facilitators to addressing FI in rural primary care practices in northern New England, USA (Maine, New Hampshire, and Vermont). A convergent parallel mixed methods design was chosen to allow for comparison and integration of quantitative and qualitative data from surveys and interviews conducted simultaneously [28]. This research was approved as an exempt study by the Dartmouth Health Institutional Review Board. Participants read a study information sheet and agreed to participate before completing the survey, and verbal consent was obtained prior to interviews. Survey incentives consisted of a $10 donation to a local food non-profit organization, and interview participants received a $50 gift card.

### Sample

Eligible respondents were medical providers or staff (such as nurses, medical assistants, care coordinators, and resource specialists) from rural primary care practices that were members of three practice research networks in northern New England, USA: The Dartmouth CO-OP Northern New England Practice-based Research Network (Dartmouth CO-OP PBRN), the Bi-State Primary Care Association, and the Northern New England Clinical and Translational Research Network. Respondents did not need to be actively involved in FI screening or interventions at their practice but were requested to have knowledge of their practice’s procedures related to screening for and addressing food insecurity among patients. Practices were categorized as rural if the practice zip code was associated with a Rural-Urban Commuting Area (RUCA) Code ≥ 4 [29]. Responses were collected at the practice level (i.e., we included only one respondent per unique practice using the practice name supplied by each survey respondent). If multiple responses were received from a practice, we included only the first response in the survey results. However, if the first respondent declined an interview but a later respondent agreed to an interview, the first survey respondent to agree to an interview was selected in order to maximize interview participation.

### Data collection

#### Survey

A web-based survey was developed by an interdisciplinary research team with expertise in primary care and public health. Survey questions focused on food security screening procedures, interventions addressing food needs, and the impact of the coronavirus disease 2019 (COVID-19) pandemic on food insecurity in the community and practice (see Supplement 1). Surveys were administered and study data were managed using REDCap electronic data capture tools hosted at Dartmouth Hitchcock [30, 31]. Survey data were collected between September 2020 and March 2021. Participants were recruited via emails to the listservs of the three participating practice networks.

#### Semi-structured interviews

Respondents who expressed interest in participating in an interview were asked to provide their contact information after the survey and were subsequently contacted by research staff via email. The research team developed a semi-structured interview guide based on preliminary survey results and existing literature on addressing FI in primary care. The interview guide included questions about barriers to food security in the community, food insecurity screening and interventions within the practice, and the response of the practice and community to food insecurity during the COVID-19 pandemic. To ensure we addressed potential barriers and facilitators across a range of implementation domains in our interviews, we mapped interview guide questions to the Consolidated Framework for Implementation Research (CFIR) [32, 33] (see Supplement 2). CFIR is a widely-used framework for planning and evaluating program implementation that includes constructs across five domains. Interviews were conducted via phone by research staff (KJ) between January and May 2021. Interviews were audio-recorded and transcribed verbatim using a professional online transcription service.

### Data analysis

Survey data were analyzed descriptively using SAS JMP Pro 15 [34]. Three research team members (KJ, MK, AS) conducted thematic analysis of interview transcripts using Dedoose Version 9.0.46 [35]. A preliminary codebook was developed based on interview content, reflection on key concepts discussed by participants, and existing literature. Three research team members (KJ, MK, AS) independently coded the same two transcripts using the preliminary codebook and discussed code application to reach consensus. The preliminary codebook was then revised based on emerging codes and discussion. The remaining transcripts were coded by pairs of researchers and reviewed with a third team member to resolve discrepancies and reach full consensus. The codebook was iteratively revised until no new codes emerged and agreement was reached among the analytic team on a final coding framework. Although our preliminary coding framework was organized by CFIR domains, [32, 33] our finalized codes did not naturally align with CFIR, and instead were organized by categories that emerged through our analysis. Once a final codebook was established, each transcript was reviewed again and discussed as a team to finalize code application. Themes and subthemes were identified based on the analytic team’s review and iterative discussion of the most common and salient ideas and meanings that emerged from coded excerpts within each category, and illustrative quotes were selected for each theme. Finally, quantitative survey results and qualitative interview themes were reviewed together to identify areas of convergence for mixed methods interpretation and data display.

## Results

### Participants

Staff and clinicians from 24 unique rural practices participated in the survey. Of those, 13 also agreed to participate in a qualitative interview. Respondent and practice characteristics are outlined in Table 1. Most respondents were clinicians, resource specialists or community health workers (CHWs), or administrators representing practices that specialize in Family Medicine, Pediatrics, and/or General Internal Medicine. The majority of practices were hospital-affiliated practices or federally qualified health centers with six or more clinicians. More than half of participating practices had a systematic process for FI screening in place.

**Table 1:** Characteristics of respondents and practices

Characteristic	Survey Practices (***n***=24) n (%)	Interview Practices (***n***=13) n (%)
**Respondent role**		
Clinician	15 (63)	5 (39)
Resource specialist/CHW	4 (17)	3 (23)
Administrator	3 (13)	3 (23)
Nurse	1 (4)	1 (8)
Care coordinator	1 (4)	1 (8)
**Practice specialty** ^**a**^		
Family Medicine	12 (50)	8 (62)
Pediatrics	9 (38)	4 (31)
General Internal Medicine	7 (29)	5 (39)
Obstetrics/Gynecology	2 (8)	2 (15)
Other	1 (4)	1 (8)
**Practice type**		
Hospital affiliated	13 (54)	6 (46)
FQHC	5 (21)	4 (31)
Private practice	3 (13)	2 (15)
Other	3 (13)	1 (8)
**Practice size**		
2-5 clinicians	4 (17)	2 (15)
6-10 clinicians	8 (33)	4 (31)
>10 clinicians	12 (50)	7 (54)
**Type of FI screening used**		
Systematic screening	13 (54)	8 (62)
Informal screening	9 (38)	5 (39)
No routine screening	1 (4)	0
Don’t know/prefer not to answer	1 (4)	0

*CHW *Community health worker, *FQHC *Federally qualified health center.

^a^More than one selection allowed

### Quantitative results

Survey results revealed several barriers to FI screening and interventions. Among practices not yet conducting systematic FI screening (*n* = 11), the most common barrier reported was time and resource constraints (91%). Frequently cited barriers to addressing FI (i.e., implementing interventions) included time and resource constraints (67%), an insufficient screening process (38%), and lack of community resources (29%). Despite barriers, almost all respondents reported that food needs were very important (71%) or somewhat important (21%) for their patients and communities.

### Qualitative results

Themes and sub-themes were organized into the major categories of Procedures, Community Factors, Patient Factors, External Factors, Practice Factors, Process and Implementation Factors, and Impact.

#### Procedures

##### Screening workflow

Several aspects of FI screening workflow and processes influenced implementation. Participants identified time limitations and competing clinical demands as significant barriers to screening. Screening processes that were simple and not time-intensive facilitated screening.

*“I think our biggest barrier is our nurses’ time… We have very high traffic clinics, and very high need populations… When we ask them to ask another question, sometimes that can really feel like a burden, understandably.”* – Administrator A.

*“I think that the process for [medical assistants] to gather the information, and then record it, is something simple and quick. So, because it doesn’t add a lot of time burden, it’s doable and sustainable.”* – Provider B.

Consistent and systematic processes using formal screening tools facilitated screening at some practices. These practices often screened routinely during certain visit types, particularly annual physical exams. Some practices used a pre-visit screening process to address the limited time in clinic. Respondents found it useful to integrate FI screening into the electronic medical record and to bundle FI screening with screening for other social determinants of health. In some practices, screening was done informally if the topic arose during a clinic visit. Informal processes typically resulted in less consistent screening.

*“It’s really sort of only if [food insecurity is] somehow identified by the physician or nurse in their interaction with the patient, so it’s not systematic at all. It’s just really fairly ad hoc and random.”* – Provider A.

Some practices used framing, or explaining the purpose of FI screening to patients, to facilitate acceptability. For example, some practices used written explanations for paper screening or verbal explanations for staff-administered screening.

*“[The screener] was tweaked, a lot of… different wording, different ways to ask questions and then at the end of the screener was a little snippet about why we’re asking and there are resources available in your community and we have specialists… And since then, I have seen a huge jump in referrals from the screener. And I think that it’s just coming from that sort of simple language… we’re not asking just to ask, we’re asking because we are recognizing that your overall health and your environment and things like that really do play a huge factor in your physical health.”* – CHW C.

##### Intervention workflow

Participants noted that having staff members whose role was dedicated to operationalizing interventions, such as CHWs or social workers, facilitated practices’ ability to address FI. These staff members helped relieve time constraints of medical and nursing staff and could specialize in the resources available to patients in the practice and community.

*“We had a nurse who at the practice did care coordination… And she was like our practice champion and is still our practice champion of being that contact person between the food bank and tracking of when the emergency food bags are getting lower. So, someone to make sure the logistics of the food bags are staying in stock. So that’s been helpful to have someone whose dedicated role is to be looking into that.”* – Provider C.

Practice staff connected patients with resources to address FI as well as other social determinants of health the patients faced. In this way, establishing an initial connection with practice staff served as a point of entry to access additional support. However, some participants noted that even after connecting with intervention staff, lack of patient follow-through remained a barrier.

*“Once they’re connected with me… I can then connect them to additional resources. So, if they get referred for one program, I can also let them know about another one that may benefit them as well. And so, I think that makes it easier and is helpful.”* – Care coordinator A.

#### Community factors

##### Access and availability

Participants noted that some communities had adequate resources while others struggled with long distances between patients and resources, lack of access to healthy foods in stores, and limited hours of food programs. Overall, communities offered a variety of food resources (e.g., food banks, mobile food drops, community supported agriculture, and soup kitchens) as well as non-food resources that addressed housing insecurity, poverty, lack of transportation, mental health, and chronic conditions. However, even when resources were available, eligibility requirements sometimes prevented patients from receiving needed support.

*“… some people have just enough family support where they can’t get a few more food stamps. Or they have ownership of their home, but they have nothing else. And so, because they have this asset, they don’t qualify for resources.” –* Provider B.

Some participants discussed food resources that are unique to their rural communities, such as local farms and access to land for gardening, which supported food access for patients.

*“I think it’s because more rural, actually there’s less barriers. There are a fair amount of my patients who also have small gardens and small farms and stuff, so I think we’re in better shape here than a lot of places.”* – Provider D.

##### Community connections

Strong connections between practices and community food programs or other organizations (such as schools) supported efforts to address FI. Some practices were involved in community coalitions, working closely alongside other organizations to address FI. Strong connections to community organizations also facilitated on-site food distribution at the practices by helping with the coordination of food distribution and providing staffing and food to distribute.

*One of our health centers that has a particularly high food insecurity rate in their community, in partnership with the Vermont Foodbank and the local school, started a food pantry say three or four years ago. It was the first in that county, in that area. They helped staff it. They’re on the volunteer board of directors, our clinic site staff, and they really work with other community partners to source food and distribute food through that food pantry.* – Administrator C.

#### Patient factors

##### Acceptability

Participants generally reported that screening and interventions were acceptable to patients, though stigma associated with FI posed a barrier. In some ways, the rural setting of the practices contributed to stigma, for example due to the lack of privacy in close rural communities. Practices worked to increase the acceptability of FI screening and interventions through strong patient-staff relationships and warm handoffs.

*“One of the things that I think is also unique to the rural setting is everybody knows everybody. I think there’s also a little bit of a factor of anonymity is lost. Sometimes people are more hesitant to share that they need assistance with food. It seems like such a basic thing, everybody kind of assumes everyone has food.”* – Administrator B.

*“I think it’s a lot to do with intention and relationship-building. Again, staff who really value that and are super patient-centered are able to build those relationships quickly and make it acceptable and okay to accept those food and that help.”* – Administrator C.

##### Context and characteristics

Participants discussed patient characteristics and contextual factors that contributed to FI and impacted practices’ ability to address FI. Related social determinants of health including poverty, housing insecurity, limited internet connectivity, and especially lack of transportation were common barriers noted. Gaps in patient knowledge including awareness of community resources and how to prepare healthy food presented barriers to patients accessing and utilizing food resources.

*“Transportation is a barrier to accessing healthy food. I guess, playing along with transportation, it’s just the distance to accessing food, even if you have transportation. So, the time commitment involved with shopping or visiting pantries.”* – Provider C.

*“The transportation’s a big thing, but I also think it’s education. I think a lot of people just don’t know what’s out there.”* – Administrator B.

Certain patient populations, including older adults, individuals with dietary restrictions (e.g., for diabetes), and individuals with mental health conditions faced additional challenges related to FI. Motivation, competing demands, and hesitancy about receiving help sometimes impacted patient engagement and prevented patients from accessing FI resources when offered.

*“… [patients] go to their doctor’s appointment and the doctor says, “You’re diabetic. We need you to start getting on a better diet.” The patient goes into the supermarket and realizes that everything that they’re supposed to buy, they can’t afford.”* – CHW A.

#### External factors

##### Endorsement by outside organization

Participants reported that endorsement of a FI or general social risk screening tool by an outside organization influenced the practice’s adoption of a specific tool.

*“There was nothing else that was ever presented which was more evidence-based or quicker, either from our partners… or the hospital here or from the state… They all endorsed the Hunger Vital Sign to question screen. So, it seemed like a no-brainer for us.”* – Administrator C.

##### External funding

Some practices benefited from external funding to support practice-based interventions or the salaries of staff members conducting interventions.

*“So, two of these programs don’t cost anything for our practice. One of them is an investment. And I think one of the resources that helps with that is grants.”* - Care coordinator A.

#### Practice factors

##### Infrastructure

Many participants described small physical spaces at practices that made providing on-site food or resources difficult. Large, rural service areas of practices made arranging food deliveries or keeping updated lists of resources challenging.

*“We have space constraints so it’s not like we could have a huge pantry with refrigerated food and produce available for people. I know that’s a barrier.”* – Administrator C.

*“We’re serving many towns and communities in the area; some are 40 minutes to an hour away even.”* – Care coordinator A.

Availability of interdisciplinary staff, including dieticians, mental health providers, case managers, and social workers, facilitated practice capacity to address FI.

*“Specifically, we have, like I said, care coordination. Those help with all sorts of things. Health insurance, food access, fuel access, all kinds of things. Then we have our chronic care management team. They work specifically with patients who have two or more chronic conditions. They work with them on creating an action plan and making lifestyle decisions. They also work closely with our care coordinators to get them connected with resources.”* – Administrator A.

##### Additional support needed

Participants identified additional support that could facilitate their practices’ ability to address FI, including support for process development and implementation, additional staff and funding, and information on community FI rates.

*“If somebody’s cracked a really awesome way of working the screening into a visit so it can happen at every visit, that would be amazing…. And how are people then also tracking the interventions and which one of those interventions are most successful? I’d be really interested in hearing from other practices that have figured that stuff out.”* – Administrator C.

##### Priority and value

Most practices placed a high priority on screening and interventions for FI, with some respondents reporting that addressing FI was aligned with the mission or philosophy of the practice. Other practices noted competing priorities and instead focused efforts on other areas, such as depression or heart disease.

*“…most of the folks who work in the clinics live in our community. They’re not just coming to work and seeing the patients and then going home. They’re coming to work and then seeing the people they know in the community, and it’s deeply important to us.”* – Administrator A.

*“…our practice really sees this as highly important, and values this work. It fits in with our practice motto or mission of really looking at patients from this whole person perspective of medical, dental, mental health. And food security is a big part of all of that. Nutrition and healthy food behaviors, all that is all encompassing, and within all those realms of the practice.” – Care Coordinator A*.

##### Internal communication and information sharing

Practice staff engaged in FI screening and interventions communicated often about FI within the clinic. Frequent communication allowed for timely feedback on screening efforts to sustain engagement by practice staff and providers.

*“I think that it is a top priority for us… we review our numbers every single month, and that has been really helpful because we work as a team and we’re a big dynamic team, so we have to have key people who go back to their pods and relay the information that we’re all discussing. I think it’s been important to A, work as a team that distributes information, and then B, stay on top of the numbers.”* – Administrator A.

#### Process and implementation factors

##### Change agents and buy-in

Internal and external change agents provided leadership and energy for initiating and sustaining FI screening and intervention processes. Change agents helped gain buy-in from practice administrators, providers, and staff responsible for screening or interventions, which was crucial for practices to sustain efforts to address FI.

*“There’s at least one or two people at each of our practices where food security is their passion. They may be working in a job that’s in a medical field, but this is why they wake up in the morning. Having those embedded champions in there, you can’t lose.”* – Administrator C.

##### Evaluation

Most practices did not have formal evaluation processes for FI screening and interventions. Practices that tracked data on screening and intervention rates were able to adjust tactics and remind staff members of screening importance.

*“So, when somebody does screen positive for food insecurity, what’s actually happening? …That’s our work this year, is to try to figure out what interventions were delivered and how useful were those. But we’re not there yet.”* – Administrator C.

##### Improvements needed and planned

Participants described several desired and/or planned improvements to screening processes, including creating a formal screening process and using the electronic medical record to track screening results. Some practices sought to increase the frequency of screening or the patient populations that were screened. For interventions, some practices hoped to increase onsite food availability.

*“Now that everything is electronic, one of our goals is to identify ways to have an official screening process for food insecurity, and to be able to somehow navigate alerting providers in the system that that is ongoing for this patient, or that they’re struggling with that currently.”* – Care coordinator A.

#### Impact

##### Positive impacts of addressing FI

Respondents described positive impacts of clinic efforts to address FI. Addressing FI had benefits for patients, including meeting food needs, improving health and quality of life, increasing awareness of resources, and instilling a sense of support from practices. Benefits to practice staff included enabling staff to assist patients and raising awareness of FI in the community.

*“Letting patients know that they’re not alone and that their medical practice can help them, I think that’s been one of the biggest accomplishments, especially in my role… So, I noticed that when patients know these services, it’s sort of like something’s been lifted off of their shoulders… It opens a lot of more avenues and patients feel that they have a safe haven.”* – CHW A.

### Integrated analysis

As shown in the joint display, survey findings on barriers to FI screening and interventions and potential facilitators aligned well with themes identified through qualitative analysis (Table 2).

**Table 2:** Mixed interpretation of barriers to FI screening and interventions from surveys and interviews

Survey Question	Survey responses n (%)	Corresponding qualitative interview themes
**Barriers to FI screening** ^**a,b**^ (*n*=11)		• Limited time in clinic to conduct screening was a barrier • Consistent and systematic process facilitated screening • Improvements needed and planned: systematic processes, increase onsite food availability
Time and resource constraints	10 (91)	
Don’t know how to implement	1 (9)	
Inability to address needs	2 (18)	
Other	1 (9)	
Don’t know/prefer not to answer	1 (9)	
**Barriers to addressing FI** ^**b**^ (*n*=24)		• Access to and availability of community resources was variable • Strong community connections facilitated addressing FI • Dedicated personnel to address needs facilitated addressing FI • Supports needed: process development and implementation, additional staff, information on community FI rates
No barriers	2 (8)	
Lack of community resources	7 (29)	
Insufficient screening process	9 (38)	
Time and resource constraints	16 (67)	
Lack of knowledge about FI	4 (17)	
Other	2 (8)	
Don’t know/prefer not to answer	1 (4)	
**Importance of food needs for patients and communities** (*n*=24)		• Most practices placed a high priority on screening and interventions to address FI • Change agents with a passion for FI efforts supported implementation and sustainment
Very important	17 (71)	
Somewhat important	5 (21)	
Neutral	2 (8)	
Not very important	0	
Not important at all	0	

^a^Due to branching logic in the survey, the question on barriers to FI screening was only asked of practices that did not have a systematic screening in place.

^b^More than one selection allowed

## Discussion

Our study identified key barriers and facilitators to addressing FI in rural primary care practices related to screening and intervention workflows, community factors, patient factors, external factors, and practice factors. Interview and survey data aligned to reveal time and resource constraints as a major barrier to screening for and addressing FI. Food needs of patients and communities were “very important” to 71% of survey respondents, and interview themes similarly revealed that most practices place a high priority on addressing FI. Interviews demonstrated the positive impacts of screening for and addressing FI among both patients and clinic staff. However, interview participants also identified several areas for improvement in their practices, including implementing formal screening processes and increasing onsite food availability.

Limited time and resources was identified as a key barrier to FI screening. Prior studies revealed time constraints as a barrier to screening for FI in primary care, [11, 12] with additional concerns about the time needed to address FI when it was identified [12]. The timing of our study during the COVID-19 pandemic likely impacted responses. The pandemic put additional strain on practices, including through the rapid adoption of telemedicine [36, 37] and increasing burnout among staff members [37]. Some practices in our sample navigated the challenge of limited time by using pre-visit screening and/or performing screening using an electronic health record. Respondents noted that additional staff were needed to support efforts to address FI, which could reduce the time barrier. A recent study found that the estimated cost to primary care practices of providing evidence-based interventions to address health-related social needs was about twice the level of federal funding provided for these services [38]. Further investment in staffing and resources to support FI screening and interventions could facilitate the development and implementation of effective practice-based FI programs.

Barriers specifically attributed to the rural setting of practices in this study included lack of transportation and large geographic service areas. The combination of limited transportation for patients and large service areas required coordination from practice staff to assist patients in distant communities and keep track of food resources in multiple areas. Patients experiencing FI have reported transportation as a barrier to food security, [39] and our team previously found that lack of transportation was a barrier to addressing FI among practices providing prenatal care in northern New England [27]. An additional barrier was the loss of patient anonymity in small rural communities, which was thought to increase feelings of stigma. Stigma has been identified as a barrier to FI screening in prior studies, [10] but it could be exacerbated by closer community relationships in the rural communities represented in our study. Practices sought to minimize stigma through strong patient-staff relationships and trust, as described as a facilitator in prior work, [10] and by framing screening questions through explanation of purpose.

Participants also identified facilitators unique to rural settings, including access to land for food production through farming and home gardening. Prior research has demonstrated the positive impact of community-supported agriculture on diet quality among socioeconomically vulnerable individuals in rural areas [24]. Participants discussed a variety of community programs that addressed gaps in food availability including food banks, mobile food drops, and soup kitchens. However, we found variability in responses about the overall availability and accessibility of community food resources. This likely reflects the unequal geographic distribution of food in rural settings [16].

Close connections between practices and community or state organizations facilitated addressing FI. This was true especially for on-site food distribution at practices. Clinic-community partnerships have resulted in successful primary care interventions to address patient food needs, including case managers who refer to food resources, assistance with benefit applications, food prescription programs, farmers market vouchers, and in-office food pantries sourcing food from community organizations [3, 40–44]. These programs have demonstrated success in connecting patients with food resources, [3] but more research is needed on the impact of such programs on health outcomes, health care utilization, and cost.

Practice staff whose role was dedicated to addressing FI facilitated practice programs, coordinated with community organizations, and increased buy-in from providers and administrators. Certain individuals had particular interests in and motivation for addressing FI and acted as practice champions. Characteristics of champions that facilitate successful practice change include engagement and credibility in intervention activities, influence with practice members, and capacity (including time) to conduct intervention activities [45]. Champions at practices in our study demonstrated these qualities and were described as key actors in practices’ ability to address FI. Identification of a practice champion could serve as a step towards implementing screening and/or interventions for FI at rural primary care practices.

Several practices that utilized informal FI screening aimed to implement a formal screening process in the future. Without formal screening, practices may have difficulty gauging community FI rates since barriers such as stigma may make patients hesitant to discuss FI. Even when formal screening was performed, practices in our sample often did not evaluate the results to determine rates of positive screening or the outcome of interventions. Formal FI screening allows for the identification and tracking of FI in a patient population. In some instances, it identifies a larger number of food insecure patients than expected by clinic staff, which can serve as a motivator for sustained screening [46]. Having a team member such as a social worker to assist patients who screen positive can make practice staff more willing to adopt formal FI screening [46]. Experience from other health systems in adopting FI screening and interventions [46] could aid clinics in launching formal FI processes, determining community FI, and identifying the most effective interventions for addressing FI.

### Limitations

We studied a small sample of respondents from primary care practices in one rural region of the U.S, which limits the generalizability of the results. The setting during the COVID-19 pandemic was a compounding factor that could have impacted our results. Recruitment was challenging as strain on primary care practices during this period was high. Reports from northern New England predating our study found that rates of FI increased during parts of the pandemic, [47] which could have led practices to focus more energy on addressing FI. We sought to distinguish the impact of the COVID-19 pandemic by asking separate questions about screening and intervention procedures during the pandemic and analyzing these data separately. We relied on the knowledge of practice staff regarding FI procedures and programs, which may vary from actual practice activities. Finally, patient perspectives were not gathered in this study and future work is needed to understand the patient experience. Despite these limitations, our study is one of the first to present mixed methods findings of barriers and facilitators to addressing FI in rural primary care practices.

## Conclusion

Our study highlights the discrepancy between the perceived importance of food needs among rural primary care practices versus the implementation of systematic screening for FI. Rural primary care providers and staff in this study highlighted the importance of food needs among their patients and the value that many practices place on identifying and addressing FI. However, they also noted barriers to implementation, most importantly limited time, resource constraints, and stigma. Despite this, many practices implemented innovative interventions within the practice (e.g., practice staff members dedicated to offering resources and connecting patients to food programs) and in partnership with community organizations (e.g., mobile food drops or food pantries hosted at practices). Unique aspects of the rural settings included geographically large practice service areas, loss of privacy in small communities, and food access through farming and gardening. Overall, initial steps to addressing FI within rural primary care practices include adopting systematic FI screening, analyzing results to determine community needs, strengthening practice-community partnerships to facilitate referrals, and identifying dedicated staff to support FI efforts. Future work is needed to identify evidence-based practices for FI screening and intervention to reduce barriers to implementation in rural primary care.

### Supplementary Information

Supplementary Material 1: Supplement 1. Survey questions: Practice-based capacity for identifying and addressing food insecurity. Survey questions.

Supplementary Material 2: Supplement 2. Food Insecurity Interview Guide. Interview guide and table with interview questions and associated Consolidated Framework for Interventions Research (CFIR) Domains.

## Data Availability

The data that support the findings of this study are available from the corresponding author upon request.

## Abbreviations

FI: Food insecurity
RUCA: Rural-Urban Commuting Area
COVID-19: Coronavirus disease 2019
CFIR: Consolidated Framework for Implementation Research
CHWs: Community health workers
