# Supplementary material for: Addressing food insecurity in rural primary care: a mixed-methods evaluation of barriers and facilitators
Source: BMC Prim Care. 2024 May 11;25:163. doi: 10.1186/s12875-024-02409-1 (PMC11088768; doi:10.1186/s12875-024-02409-1)
Supplement: Supplementary file 1 — Supplementary Material 1: Supplement 1. Survey questions: Practice-based capacity for identifying and addressing food insecurity. Survey questions. [file 12875_2024_2409_MOESM1_ESM.docx]

**Supplement 2. Food Insecurity Interview Guide**

*Interview questions related to the COVID-19 pandemic were previously included as supplementary material in: Suresh A, Jordanova KE, Boardman MB, Canavan CR, D'cruze TT, Dev A, Kennedy MA. "Everyone's struggling right now": Impact of COVID-19 on addressing food insecurity in rural primary care. Fam Pract. 2023 Nov 14:cmad105. doi: 10.1093/fampra/cmad105. Epub ahead of print. PMID: 37962422.*

*These questions will guide the interview but may be adjusted as needed during the interview process.*

1. Please describe your role in your practice.

**Barriers to Food Security**

1. What are some barriers in your community that could lead patients to experience food insecurity?
   1. *For Rural practices only:* Are there any factors related to your practice’s rural setting?
2. What barriers do your clinic’s patients have in accessing resources to help with food insecurity?

**Food Insecurity Screening**

1. What are your practice’s processes for identifying food insecurity in general?
   1. How were these processes developed?
   2. Was any evidence used to develop or choose the screening process?
   3. How difficult was the process to implement?

*If participant’s practice does not screen for food insecurity AT ALL (i.e. no formal or informal screening), skip to question 8. Ask questions 5-7 if practice conducts any type of screening, including informal.*

1. What has helped your practice implement and sustain these processes?
   1. What about your practice environment has helped?
      1. The staff?
      2. The workflow?
   2. Have any characteristics or attitudes of your patient population helped?
   3. Are there any external policies or community factors that have helped?
2. What barriers has your practice faced in implementing food insecurity screening?
   1. Any challenges related to your practice environment?
      1. The staff?
      2. The workflow?
   2. Any challenges related to characteristics or attitudes of your patient population?
   3. Any challenges related to external policies or community factors?
3. Are there any improvements to your practice’s screening procedures you can imagine?

*Questions 8 – 10 are for participants whose practice does not conduct formal screening for food insecurity. Ask if practice does not screen or only screens informally.*

1. Has your practice discussed adopting a formal screening process for food insecurity?
2. What do you think are the main reasons your practice does not have a formal screening process for food insecurity?
   1. Any challenges related to your practice environment?
      1. The staff?
      2. The workflow?
   2. Any challenges related to your patient population?
   3. Any challenges related to external policies or community factors?
3. What would need to change in order for your practice to adopt a formal screening process for food insecurity?
   1. Change in workflow?
   2. Change in available resources?

**Food Insecurity Interventions**

1. What kinds of interventions does your practice offer patients for food insecurity?
   1. Any resources or interventions within the clinic?
   2. Any linkages to community resources outside the clinic?
   3. How connected is your practice to the community resources?
   4. Are there any resources that would be helpful for patients but are not available?
2. What has helped your practice implement and sustain these processes?
   1. What about your practice environment has helped?
      1. The staff?
      2. The workflow?
   2. Have any characteristics or attitudes of your patient population helped?
   3. Are there any external policies or community factors that have helped?
3. What barriers has your practice faced in implementing food insecurity interventions?
   1. Any challenges related to your practice environment?
      1. The staff?
      2. The workflow?
   2. Any challenges related to characteristics or attitudes of your patient population?
   3. Any challenges related to external policies or community factors?
4. Can you describe any goals or benchmarks your clinic has to address food insecurity?

**COVID-19 Response**

1. Have you noticed any changes in your patient population regarding food insecurity since the start of the COVID-19 pandemic?
   1. More discussions about food insecurity in clinic?
   2. More patients seeking referral to community resources?
   3. Any change in perspectives or behaviors of your patient population?
2. How has your practice responded to food insecurity during the COVID-19 pandemic?
   1. By changing screening practices?
   2. By changing interventions?
   3. What strategies have worked well?
   4. What strategies have not worked well?
   5. What changes could be made to improve the strategies?
3. How has your practice’s relationship with community organizations that address food insecurity changed as a result of the COVID-19 pandemic?
4. Relationships with existing partners?
5. New partnerships?
6. Is there any support that would be helpful for the clinic to address food insecurity going forward?
7. Is there anything else you would like to add about how your practice addresses food insecurity?

**Interview Guide with Consolidated Framework for Intervention Research (CFIR) Domains**

| Question | Probes | Notes | CFIR Domain |
| --- | --- | --- | --- |
| 1. Please describe your role in your practice. |  |  |  |
| TOPIC: BARRIERS |  |  |  |
| 2. What are some barriers in your community that could lead patients to experience food insecurity? | *For Rural practices only:* Are there any factors related to your practice’s rural setting? |  | Outer Setting – Patient Needs & Resources |
| 3. What barriers do patients of your clinic have in accessing resources to help with food insecurity? |  |  | Outer Setting – Patient Needs & Resources |
| TOPIC: SCREENING |  |  |  |
| 4. What are your practice’s processes for identifying food insecurity in general? | How were these processes developed?  Was any evidence used to develop or choose the screening process?  How difficult was the process to implement? |  | Intervention Characteristics  - Intervention Source  - Evidence Strength and Quality  - Complexity |
| 5. What has helped your practice implement and sustain these processes? | What about your practice environment has helped?  - staff  - workflow  Have any characteristics or attitudes of your patient population helped?  Are there any external policies or community factors that have helped? |  | Inner Setting – Networks & Communications, Implementation Climate  Outer Setting – Patient Needs & Resources  Outer Setting – External Policy & Incentives |
| 6. What barriers has your practice faced in implementing food insecurity screening? | Any challenges related to your practice environment?  - The staff?  - The workflow?  Any challenges related to characteristics or attitudes of your patient population?  Any challenges related to external policies or community factors? |  | Inner Setting  - Networks & Communications  - Implementation Climate  Outer Setting – Patient Needs & Resources  Outer Setting – External Policy & Incentives |
| 7. Are there any improvements to your practice’s screening practices you can imagine? |  |  | Intervention Characteristics – Relative Advantage |
| 8. Has your practice discussed adopting a formal screening process for food insecurity? |  | *Practices that do not screen only* | Implementation Climate  - Compatibility  - Tension for Change  - Relative Priority |
| 9. What do you think are the main reasons your practice does not have a forma screening process for food insecurity? | Any challenges related to your practice environment?  - The staff?  - The workflow?  Any challenges related to your patient population?  Any challenges related to external policies or community factors? | *Practices that do not screen only* | Inner Setting  - Networks & Communications  - Implementation Climate  Outer Setting – Patient Needs & Resources  Outer Setting – External Policy & Incentives |
| 10. What would need to change in order for your practice adopt a formal screening process for food insecurity? | Change in workflow?  Change in available resources? | *Practices that do not screen only* | Inner Setting – Networks & Communications, Implementation Climate  Outer Setting – Cosmopolitanism |
| TOPIC: INTERVENTION |  |  |  |
| 11. What kinds of interventions does your practice offer patients for food insecurity? | Any resources or interventions within the clinic?  Any linkages to community resources outside the clinic?  How connected is your practice to the community resources?  Are there any resources that would be helpful for patients but are not available? |  |  |
| 12. What has helped your practice implement and sustain these processes? | What about your practice environment has helped?  - The staff?  - The workflow?  Have any characteristics or attitudes of your patient population helped?  Are there any external policies or community factors that have helped? |  | Inner Setting – Networks & Communications, Implementation Climate  Outer Setting – Patient Needs & Resources  Outer Setting – External Policy & Incentives |
| 13. What barriers has your practice faced in implementing food insecurity interventions? | Any challenges related to your practice environment?  - The staff?  - The workflow?  Any challenges related to characteristics or attitudes of your patient population?  Any challenges related to external policies or community factors? |  | Inner Setting – Networks & Communications, Implementation Climate  Outer Setting – Patient Needs & Resources  Outer Setting – External Policy & Incentives |
| 14. Can you describe any goals or benchmarks your clinic has to address food insecurity? |  |  | Implementation Climate  - Goals and Feedback  - Tension for Change  - Relative Priority |
| TOPIC: COVID RESPONSE |  |  |  |
| 15. Have you noticed any changes in your patient population regarding food insecurity since the start of the COVID-19 pandemic? | More discussions about food insecurity in clinic?  More patients seeking referral to community resources?  Any change in perspectives or behaviors of your patient population? |  | Outer Setting – Patient Needs & Resources |
| 16. How has your practice responded to food insecurity during the COVID-19 pandemic? | By changing screening practices?  By changing interventions?  What strategies have worked well?  What strategies have not worked well?  What changes could be made to improve the strategies? |  |  |
| 17. How has your practice’s relationship with community organizations that address food insecurity changed as a result of the COVID-19 pandemic? | Relationships with existing partners?  New partnerships? |  | Outer Setting - Cosmopolitanism |
| 18. Is there any support that would be helpful for the clinic to address food insecurity going forward? |  |  |  |
| 19. Is there anything else you would like to add about how your practice addresses food insecurity? |  |  |  |
