# Supplementary material for: Addressing food insecurity in rural primary care: a mixed-methods evaluation of barriers and facilitators
Source: BMC Prim Care. 2024 May 11;25:163. doi: 10.1186/s12875-024-02409-1 (PMC11088768; doi:10.1186/s12875-024-02409-1)
Supplement: Supplementary file 2 — Supplementary Material 2: Supplement 2. Food Insecurity Interview Guide. Interview guide and table with interview questions and associated Consolidated Framework for Interventions Research (CFIR) Domains. [file 12875_2024_2409_MOESM2_ESM.docx]

**Supplement 1. Survey Questions: Practice-based capacity for identifying and addressing food insecurity**

*Survey questions related to the COVID-19 pandemic were previously included as supplemental material in: Hatchell KE, Canavan CR, D'cruze T, Suresh A, Dev A, Boardman M, Kennedy MA. The Impact of the COVID-19 Pandemic on Food Insecurity in Northern New England Primary and Prenatal Care Settings. J Prim Care Community Health. 2022 Jan-Dec;13:21501319221106626.*

1. Practice & respondent information

| 1.1.0 | Date of survey | Mm/dd/yyyy |  |
| --- | --- | --- | --- |
| 1.2.0 | Respondent role | 1, provider (i.e. physician, nurse practitioner, or physician assistant)  2, clinical nurse (RN, LPN)  3, medical assistant  4, care coordinator  5, social worker  6, resource specialist or community health worker  7, administrator  8, other |  |
| 1.3.0 | Name of practice: |  |  |
| 1.4.0 | Practice specialty  *Select all that apply* | 1, pediatrics  2, obstetrics and gynecology  3, family medicine  4, general internal medicine  5, other |  |
| 1.4.1 | Other practice specialty, please specify |  | 1.4.0=5 |
| 1.4.2 | Practice type | 1, hospital-affiliated  2, Federally Qualified Health Center (FQHC)  3, private practice  4, other |  |
| 1.4.3 | Practice size | 1, solo practitioner  2, 2-5 providers  3, 6-10 providers  4, More than 10 providers |  |
| 1.5.0 | Practice state |  | Select from list |
| 1.5.1 | Practice zip code |  | 5-digit |

2. Current food security screening practices

| 2.1.0 | How is food security assessed at your practice? | 1, we do not routinely assess food security status  2, informally through patient dialogue (no formal screening tool)  3, systematic screening process  99, don’t know or prefer not to answer | If 1 or 2 or 99🡪 skip to 2.8.0  *Required |
| --- | --- | --- | --- |
| 2.1.1 | Does your practice use a specific food security screening tool and/or a general social needs screening tool to screen for food security?  *Select all that apply* | 1, specific food security screening tool  2, general social needs screening tool  99, don’t know or prefer not to answer | 2.1.0=3  *Required |
| 2.2.0 | What specific food security screening tool is used?  *Select all that apply* | 1, Hunger Vital Sign (2 items)  2, USDA US Household Food Security Survey (18 items)  3, USDA US Adult Food Security Module (10 items)  4, USDA Short Form Food Security Module (6 items)  5, Baer two-item food insecurity screen (2 items)  6, Brief Hunger Screening Tool (1 item)  7, Food Security Survey Module for Youths (9 items)  8, Practice/hospital developed food-specific tool  9, Other  99, Don’t know or prefer not to answer | 2.1.1=1  *Required |
| 2.2.1 | Other specific food security screening tool, please specify: |  | 2.2.0=9 |
| 2.2.3 | What social needs screening tool is used?  *Select all that apply* | 1, Accountable Health Communities (AHC)/CMS (10+ items)  2, PRAPARE (17 items)  3, Health Leads (10 items)  4, HealthBegins (24 items)  5, WellRx (10 items)  6, Institute of Medicine (IOM)/NAM domains (12+ items)  7, WE CARE (12 items)  8, Child Poverty Tool & Resource Guide (CPTRG) (7 items)  9, IHELP (13 items)  10, Epic Social Determinants of Health module  11, Practice/hospital developed social needs tool  12, Other  99, don’t know or prefer not to answer | 2.1.1=2  *Required |
| 2.2.4 | Other social needs screening tool, please specify |  | 2.2.3=12 |
| 2.3.0 | How is food security screening administered?  *Select all that apply* | 1, self administered, electronic (tablet or through EMR)  2, self administered, paper  3, verbal screening with clinic staff  4, other  99, don’t know or prefer not to answer | 2.1.0=3  *Required |
| 2.4.0 | Who is screened for food security? | 1, all patients  2, only patients meeting certain criteria (e.g. certain appointment types, age range, etc.)  99, don’t know or prefer not to answer | 2.1.0=3  *Required |
| 2.4.1 | Please explain which patients are screened. |  | 2.4.0=2 |
| 2.5.0 | Is food security status documented in patient medical records? | 1, yes  0, no  99, don’t know or prefer not to answer | 2.1.0=3  *Required |
| 2.6.0 | How often is food security screening conducted? | 1, annually  2, every visit  3, other  99, don’t know or prefer not to answer | 2.1.0=3  *Required |
| 2.7.0 | Who is responsible for reviewing food security screening results?  *Select all that apply* | 1, provider (i.e. physician, nurse practitioner, or physician assistant)  2, clinical nurse (RN, LPN)  3, medical assistant  4, care coordinator  5, social worker  6, resource specialist or community health worker  7, administrator  8, other  99, don’t know or prefer not to answer | 2.1.0=3  *Required |
| 2.8.0 | Is your practice interested in formal screening for food insecurity? | 1, yes  0, no  99, don’t know or prefer not to answer | 2.1.0=1,2,99  *Required |
| 2.8.1 | What barriers are there for your practice to implement formal food security screening?  *Select all that apply* | 1, time and resource constraints of providers and staff  2, don’t know how to implement screening  3, inability to address food needs once identified  4, other  99, don't know or prefer not to answer | 2.1.0=1,2,99  *Required |
| 2.8.2 | What other barriers are there? |  | 2.8.1=4 |

3. Addressing food needs

| 3.1.0 | How does your practice address food insecurity?  *Select all that apply* | 1, facilitate referral to community resources  2, provide list of community resources  3, connect with practice-based social worker, resource specialist, or community health worker  4, onsite food provision or food prescription  5, food prescription for offsite food support  6, grow food onsite  7, culinary medicine (e.g. cooking classes)  8, no mechanisms in place  9, other  99, don’t know or prefer not to answer | *Required |
| --- | --- | --- | --- |
| 3.1.1 | Other ways food insecurity is addressed, please specify: |  | 3.1.0=9 |
| 3.2.0 | In general, what barriers does your practice experience in addressing food insecurity?  *Select all that apply* | 1, no barriers  2, lack of community resources  3, insufficient screening process  4, time and resource constraints of providers and staff  5, lack of knowledge about food insecurity  6, other  99, don’t know or prefer not to answer | *Required |
| 3.2.1 | Other barriers in addressing food insecurity, please specify: |  | 3.2.0=6 |
| 3.3.0 | How important do you think food needs are for your patients and community? | 1, very important  2, somewhat important  3, neutral  4, not very important  5, not at all important  99, don’t know or prefer not to answer | *Required |

4. Food insecurity during COVID-19 pandemic

| 4.1.0 | How would you estimate food insecurity has changed among patients in your practice since the COVID-19 pandemic began? | 1, no change  2, decrease  3, small increase  4, large increase  99, don’t know or prefer not to answer | *Required |
| --- | --- | --- | --- |
| 4.2.0 | Prior to the COVID-19 pandemic, how confident were you that your practice could address food insecurity? | 1, very confident  2, somewhat confident  3, neutral  4, not very confident  5, not confident at all  99, don’t know or prefer not to answer | *Required |
| 4.2.1 | Since the COVID-19 pandemic began, how confident are you that your practice can address food insecurity? | 1, very confident  2, somewhat confident  3, neutral  4, not very confident  5, not confident at all  99, don’t know or prefer not to answer | *Required |
| 4.3.0 | Prior to the COVID-19 pandemic, how confident were you that your community’s resources could address food insecurity? | 1, very confident  2, somewhat confident  3, neutral  4, not very confident  5, not confident at all  99, don’t know or prefer not to answer | *Required |
| 4.3.1 | Since the COVID-19 pandemic began, how confident are you that your community’s resources can address food insecurity? | 1, very confident  2, somewhat confident  3, neutral  4, not very confident  5, not confident at all  99, don’t know or prefer not to answer | *Required |
| 4.4.0 | Are you aware of new programs or services to address food insecurity since COVID-19?  *Select all that apply* | 1, yes – based at our practice  2, yes – based in the community  3, no  99, don’t know or prefer not to answer | *Required |
| 4.4.1 | What new services are available in your community or practice to address food insecurity during COVID-19?  *Select all that apply* | 1, expansion or changes to school lunch program  2, expansion or changes to meals on wheels  3, new or changed food drives or food shelves  4, donated meals from local restaurants  5, special meals or groceries for health workers  6, practice-based food program (e.g. food shelf at the practice)  7, others  99, don’t know or prefer not to answer | 4.4.0=1,2  *Required |
| 4.4.2 | Are you aware of any programs or resources that have been reduced or closed since COVID-19 began?  *Select all that apply* | 1, yes – based at our practice  2, yes – based in the community  3, no  99, don’t know or prefer not to answer | *Required |
| 4.5.0 | Please share any other comments about the impact of COVID-19 on food security at your practice and in your community. |  | Text para |

5. Interview

| 5.1.0 | May we contact you in the next few months about participating in an in-depth interview about food security in your practice and community? (If yes or maybe, you will be asked for your contact information). | 1, yes  2, maybe  3, no | If 3, end survey.  *Required |
| --- | --- | --- | --- |
| 5.1.1 | First name |  | 5.1.0=1,2 |
| 5.1.2 | Last name |  | 5.1.0=1,2 |
| 5.1.3 | Contact phone number |  | 5.1.0=1,2  9 digits |
| 5.1.4 | Contact email |  | 5.1.0=1,2  Email |
